# Bioprotection of the built environment and cultural heritage

**DOI:** 10.1111/1751-7915.12750

**Published:** 2017-07-24

**Authors:** Geoffrey Michael Gadd, Thomas D. Dyer

**Affiliations:** ^1^ Geomicrobiology Group School of Life Sciences University of Dundee Dundee DD1 5EH United Kingdom; ^2^ Division of Civil Engineering School of Science and Engineering University of Dundee Dundee DD1 4HN United Kingdom

## Abstract

The growth of microbial biofilms and various biomineralization phenomena can lead to the formation of stable layers and veneers on rocks known as ‘rock varnishes’ that can stabilize surfaces and protect from further weathering. This article describes the potential application of fungal systems for bioprotection of rock and mineral‐based substrates and the evidence to support this concept of utilizing natural or engineered colonization and metabolic properties of fungi, including lichens.

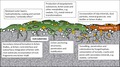

## Sustainability in the built environment

Sustainability in the built environment includes a range of concepts including selection of low environmental impact materials, design of structures whose impact during service is minimal and a planned end‐of‐life which minimizes waste. A fundamental element is ensuring that structures remain serviceable throughout their intended lives with minimal maintenance. In the case of historic buildings, this idea has been extended to the concept of ‘sustainable preservation’, which realizes environmental and cultural benefits through preservation and conservation (National Trust for Historic Preservation 2014).

Durability of buildings is dependent on the surrounding environment, with threats to resilience frequently coming from climatic conditions, substances in a building's immediate vicinity and biodeterioration by living organisms. Thus, the materials which make up the outer skin of a building determine its ability to resist deterioration. A wide variety of such materials are encountered. The nature of these materials has changed over time, but the majority are still rock‐ or mineral‐based.

Stone masonry has historically been a major component in the outer fabric of structures. Types of stone, however, vary widely, with local geology usually being the main influencing factor. From a weathering perspective, it is common to categorize stone in terms of its siliceous or calcareous nature. Calcareous stone is typically more vulnerable to deterioration under acidic conditions than siliceous stone. Another characteristic that plays an important role in determining stone durability is porosity which determines the ingress of damaging substances.

Fired clay ceramics, such as bricks and roof tiles, also have a long history as building materials. While many types of clay can be used to make these products, their chemical composition remains relatively similar – partly vitrified aluminium and silicon oxides. While fired clay is resistant to chemical attack, its porous nature means that weathering from frost and salts is a possibility.

Both stone and brick masonry are typically held together with mortar. This material normally comprises sand mixed with cement. Cements in early mortars were usually limes, but more recently Portland cement‐based mortar has become the norm. Despite this change, mortar can broadly be viewed as being comparable to calcareous stone.

In the 19th century, the possibility of using formulations similar to mortar as a bulk construction material – concrete – was revisited from its ancient Roman origins. A major step in the development of contemporary concrete was the realization that embedding steel reinforcement increased tensile strength, which widened the variety of structural forms that could be built. However, this development introduced a vulnerability because of the propensity of steel to corrode. Steel is normally protected by the alkaline conditions in concrete, but the ingress of certain substances – specifically chloride and atmospheric carbon dioxide – can override this.

The materials described above are not necessarily the final boundary between a building and the external environment. It has long been common practice to apply protective coatings to the exterior of buildings. Such coatings have historically taken the form of lime‐based whitewashes, lime and cement‐based renders and paints. Coating technologies have more recently introduced formulations with functions tailored towards specific applications. Many of these coatings are optically clear, with functions including an ability to penetrate into the substrate, impart hydrophobicity and permit water vapour movement while resisting water ingress. While these new materials have the potential to enhance durability, their use on historic buildings is not encouraged as a result of uncertainties with regard to long‐term effects (Douglas‐Jones *et al*., 2016). There is therefore a need to consider additional environmentally‐friendly approaches for protection of outer layers, with a biogenic approach appearing to offer some unique benefits.

## Microorganisms on rock and mineral‐based substrates

All rock and mineral‐based substrates, whether in the natural environment or as components of the built environment or cultural heritage, are rapidly colonized by microorganisms (Gadd, 2017a,b). Stone‐inhabiting microbes may grow on the surface (epilithic), in crevices and fissures (chasmolithic), or may penetrate some millimetres or even centimetres into the rock pore system (endolithic). Organisms may scavenge nutrients from the atmosphere and rainwater, and also use residues on surfaces, waste products of other microbes, decaying plants and insects, dust particles, aerosols and animal faeces. Bacteria, cyanobacteria, algae and fungi (including lichens) cause a range of effects including discolouration, staining and biofouling to structural and chemical alteration of the substrate. Biofilms are particularly evident in altering the appearance of stone structures, with fungi considered to be the most important chemoorganotrophs. ‘Greening’ of surfaces may result from photosynthetic cyanobacteria or algae, while ‘blackening’ may result from growth of many rock‐inhabiting fungi. Fungi and algae are probably the most obvious manifestations of microbial colonization of human‐built structures (Gadd, 2017a). Lichens are a fungal growth form, consisting of a symbiotic partnership between a fungus and a photosynthetic organism, either an alga or a cyanobacterium and sometimes both. It is now known they can also contain a yeast as another fungal partner (Spribille *et al*., 2016).

Biodeteriorative processes involve a combination of physical and biochemical mechanisms, including penetration into cracks and pores, expansion or contraction of biomass and the excretion of mineral solubilizing metabolites (Gadd, 2017a,b). Conversely, the growth of biofilms and various biomineralization phenomena can lead to the formation of stable layers and veneers on rocks known as ‘rock varnishes’ that can stabilize surfaces and protect from further weathering. These layers are not unlike some of the protective coatings applied to the exterior of buildings.

## Bioprotection of rock‐ and mineral‐based substrates

It is plausible that the bioengineered production of stable microbially mediated rock varnishes can provide a biotechnological solution for the bioprotection of surfaces in the built environment. Such protection would have the additional benefit of being more compatible with the preservation of historic buildings in comparison to commercial protective coatings.

**Figure 1:**
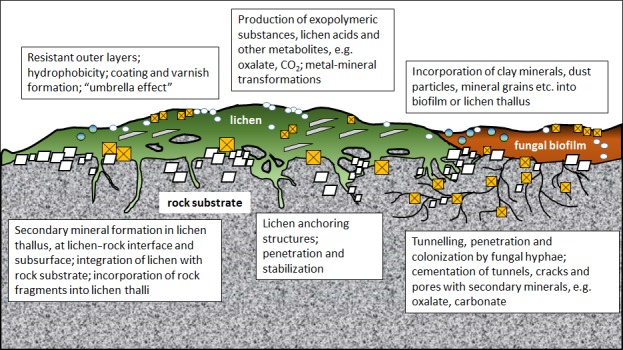
Representation of possible mechanisms involved in surface colonization of rock‐ and mineral‐based substrates by lichens and fungal biofilms. There may be wide variations in the nature of mechanisms involved depending on the lichen species, substrate and environmental factors. 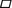, mineral formation, e.g. carbonate, in lichen thallus, rock interface or subsurface; 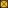, oxalate formation, e.g. calcium oxalate, in lichen thallus, rock interface or subsurface; 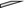, clay minerals; 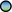, other minerals, e.g. oxides; 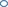, miscellaneous dust particles, mineral grains.

Fungal systems are probably of most potential when considering bioprotection approaches, and there is considerable evidence to support this concept of utilizing natural or engineered colonization and metabolic properties of fungi, including lichens. Indeed, the accelerated deterioration of building stone and cultural heritage that can occur when such protective layers are removed is well known (McIlroy de la Rosa *et al*., 2012; Gadd, 2017a). The bioprotective effect may take two forms – physical protection and biomineralization. Lichens, fungal biofilms and coatings may physically inhibit weathering of surfaces through shielding by the thallus (‘umbrella effect’), binding of the rock surface by their anchoring structures or hyphae and through the accumulation of detached fragments in lichen thalli (Banfield *et al*., 1999; Carter and Viles, 2005). Biomineralization can result from oxidation or reduction of a metal species. Additionally, the release of metals from rocks in mobile forms combined with reaction with reaction with metabolites can result in a variety of secondary mineral precipitates including carbonates, phosphates, oxides and oxalates (Gadd, 2010; Gadd *et al*., 2014). Such biomineral formations contribute to rock coating development (Gadd, 2007, 2017b; Gorbushina, 2007; Fomina *et al*., 2010) (Fig. 1).

Some lichens (especially crustose species) are epilithic (surface dwellers) and/or endolithic (interior dwellers; Wierzchos *et al*., 2012). Several of these lichens form a crust on and/or beneath the rock surface and become fully integrated within the rock substrate (Chen *et al*., 2000; de los Ríos *et al*., 2002). Endolithic lichens have been shown to protect Carboniferous limestone surfaces from weathering by rainwater (McIlroy de la Rosa *et al*., 2014). Pores in the limestone become filled with a network of hyphae which waterproof the stone and act as a barrier to sulfate ingress. A similar endolithic layer from ancient lichen growth was also found on historic monuments, possibly explaining their well‐preserved state (Concha‐Lozano *et al*., 2012). A biologically initiated rock crust on sandstone formed by various fungi, and including lichens and green algae, was enriched in kaolinite and quartz. This crust was up to 12 times less erodible and possessed a tensile strength 3–35 times higher, than the subsurface sandstone. The saturated hydraulic conductivity was 15–300 times lower than the subsurface and also decreased capillary water absorption. A major contribution to surface hardening was provided by organic matter (Slavík *et al*., 2017).

Calcium oxalates (whewellite and weddellite) occur widely in patinas on stone buildings, monuments, plasters, cave and wall paintings and sculptures (see Gadd *et al*., 2014). These calcium oxalate films are very stable and protect the underlying substrate (Del Monte *et al*., 1987). They may also contain calcium carbonate and are sometimes intermixed with lichen fragments (Del Monte *et al*., 1987). Other black crusts found on stone and mortar surfaces exposed to the atmosphere almost always contain oxalate (see Gadd *et al*., 2014). A fungal‐derived copper‐oxalate patina was also used for bioprotection of a copper artefact (Joseph *et al*., 2012).

Carbonates and oxides may also be relevant to bioprotection. Several fungi promote Mn(II) oxidation to black Mn(IV)O_2_. Manganese (and iron) oxides are major components (20–30%) along with clay (~60%) and various trace elements in rock varnish. (Gorbushina, 2007). Many microorganisms can precipitate carbonates, and precipitation of secondary calcite after fungal dissolution of limestone can result in cementation of the original limestone substrate (Verrecchia *et al*., 1990). In microbially induced carbonate precipitation (MICP), urea hydrolysis leads to increased alkalinity and precipitation of calcium or other metal carbonates (Kumari *et al*., 2016). Urease‐based MICP has been applied to enhance the durability of structures by reducing water permeation and corrosion (De Muynck *et al*., 2010; Phillips *et al*., 2013), for cementation of cracks and fissures (Ramachandran *et al*., 2001; Van Tittelboom *et al*., 2010) and the restoration of historic monuments (Tiano *et al*., 1999).

## Conclusions

Currently, the bioprotection of buildings by lichen can be considered to be a happy accident. For it to be actively employed with confidence would require a number of uncertainties to be resolved.

One area of uncertainty is how different lichen species will respond to a given substrate. The chemical and mineralogical composition of a stone substrate appear to have a strong influence over colonization and the substances that lichens produce (Adamo and Violante, 2000; de los Ríos *et al*., 2002), and hence, the biomineralization processes that can occur. This is important, because the nature of biomineralization can have significant impacts on the integrity of the material beneath. For instance, when oxalic acid is produced by lichens growing on a limestone substrate, highly insoluble calcium oxalate is precipitated which tends to seal porosity and make the underlying material less permeable. However, if this compound is produced on other substrates, it may have a less desirable effect. Moreover, lichen can produce a range of other compounds, including the lichen acids (mainly polyphenolic compounds; Adamo and Violante, 2000) whose effect on durability is less well understood. Hence, a greater understanding of which lichen species are most appropriate for a specific building material from a biomineralization perspective is needed to inform selection for bioprotective applications.

Another potential barrier is the notoriously unpredictable nature of lichen growth. In some cases, stone surfaces can be rapidly colonized by lichens, but in other instances, colonies may remain essentially the same size for tens of years. If lichens are to be used as a means of protection, strategies for maximizing the initial rate of colonization and subsequent growth must be developed. These are likely to include selection of more rapid geoactive species, techniques for inoculating surfaces and additional nutrients applied to surfaces during or after inoculation to promote growth. Despite these barriers, the low resource inputs and lack of pollutants associated with bioprotection of the built environment and cultural heritage by lichens offer a genuinely sustainable solution. Furthermore, the barriers discussed above are unlikely to be insurmountable – certainly research into using bacteria for carbonate‐mediated biorepair has made much progress in recent years (De Muynck *et al*., 2010). In many respects, lichens lend themselves more to this form of bioprotection than bacteria, given their ubiquity on all kinds of surfaces in the built environment and their physical and chemical properties as geoactive agents.

## Conflict of interest

None declared.
